# The prelimbic cortex uses higher-order cues to modulate both the acquisition and expression of conditioned fear

**DOI:** 10.3389/fnsys.2014.00235

**Published:** 2015-01-12

**Authors:** Melissa J. Sharpe, Simon Killcross

**Affiliations:** School of Psychology, University of New South WalesSydney, NSW, Australia

**Keywords:** prelimbic cortex, fear conditioning, context, extinction, learning, fear expression, bi-conditional discrimination, infralimbic cortex

## Abstract

The prelimbic (PL) cortex allows rodents to adapt their responding under changing experimental circumstances. In line with this, the PL cortex has been implicated in strategy set shifting, attentional set shifting, the resolution of response conflict, and the modulation of attention towards predictive stimuli. One interpretation of this research is that the PL cortex is involved in using information garnered from higher-order cues in the environment to modulate how an animal responds to environmental stimuli. However, data supporting this view of PL function in the aversive domain are lacking. In the following experiments, we attempted to answer two questions. Firstly, we wanted to investigate whether the role of the PL cortex in using higher-order cues to influence responding generalizes across appetitive and aversive domains. Secondly, as much of the research has focused on a role for the PL cortex in performance, we wanted to assess whether this region is also involved in the acquisition of hierarchal associations which facilitate an ability to use higher-order cues to modulate responding. In order to answer these questions, we assessed the impact of PL inactivation during both the acquisition and expression of a contextual bi-conditional discrimination. A contextual bi-conditional discrimination involves presenting two stimuli. In one context, one stimulus is paired with shock while the other is presented without shock. In another context, these contingencies are reversed. Thus, animals have to use the present contextual cues to disambiguate the significance of the stimulus and respond appropriately. We found that PL inactivation disrupted both the encoding and expression of these context-dependent associations. This supports a role for the PL cortex in allowing higher-order cues to modulate both learning about, and responding towards, different cues. We discuss these findings in the broader context of functioning in the medial prefrontal cortex (PFC).

## Introduction

Previous research has demonstrated that the prelimbic (PL) cortex facilitates the ability of animals to respond adaptively under changing circumstances. For example, function in the PL cortex is necessary to allow animals to exhibit an instrumental response which is reflective of the current value of a goal, a switch between response and strategy sets, the use of contextual cues to resolve response conflict, and a change in the degree of attention directed towards a stimulus on the basis of how well it predicts a motivationally-significant outcome (Balleine and Dickinson, 1998; Ragozzino et al., 2003; Marquis et al., 2007; Floresco et al., 2008; Sharpe and Killcross, 2014). One interpretation of these data is that the PL cortex is involved in using higher-order information to influence a response. More specifically, we would argue that the PL cortex facilitates a process whereby contextual cues, goal value, the predictive history of a stimulus, or a strategy set, exert top-down control over the ability of a particular stimulus to elicit a behavioral response. This allows the PL cortex to contribute to a process where animals can use information in the environment to exhibit flexible behavior.

A finding that illustrates this concept well is the demonstration that PL inactivation disrupts the ability of animals to use contextual cues to resolve response conflict in a rodent version of the Stroop task (Marquis et al., 2007). In this task, rats are trained on two bi-conditional discriminations, one auditory and one visual, in two distinct contexts. In one context, the two visual cues dictated pressing either the left or right lever, and in the other context the auditory cues would dictate the correct lever press. The PL cortex was inactivated before a test session where animals were presented with two types of novel audio-visual compounds, congruent and incongruent, in both contexts. Congruent compounds comprised two stimuli that dictated the same lever press during training, whereas incongruent compounds comprised stimuli that dictated opposing lever presses during training. On incongruent compound trials, animals needed to use the task-setting contextual cues in order to disambiguate response conflict and perform the correct lever press (i.e., the lever press trained in the test context). Inactivation of the PL cortex before test specifically disrupted performance on the incongruent trials. These data demonstrate that the PL cortex is necessary to allow animals to use the contextual cues to disambiguate the response conflict and perform the lever response that was trained in the test context. In terms of the framework described above, we may view the PL cortex as facilitating a top-down influence of the contextual cues present during the test session to bias performance of the response associated with the stimulus trained in that context.

Despite the wealth of evidence which demonstrates that the PL cortex is involved in facilitating the ability of animals to respond flexibly in complex environments in appetitive procedures, there is a relative paucity of such research in the aversive domain. However, recently data has begun to emerge to suggest that the PL cortex may be involved in the ability of animals to use contextual cues to influence responding in fear conditioning procedures. For example, Orsini et al. (2011) found that disconnection of the hippocampus and the PL cortex disrupted the ability of animals to exhibit renewal of conditioned fear following extinction. Orsini et al. (2011) paired a CS with shock in one context (context A), extinguished the CS in an alternative context (context B), and tested animals for levels of fear expressed towards the CS in another familiar context (context C; i.e., an ABC renewal paradigm). Under normal circumstances, rats will again express fear when they are placed in context C. This effect has been argued to be due to the context dependence of fear learning following extinction (Bouton, 1993, 1994). That is, when the CS is presented in a different context following conditioning in the absence of shock, animals use the contextual cues to disambiguate the significance of the CS as it now has multiple meanings (Bouton, 1993, 1994; Harris et al., 2000). Consequently, it is argued that animals form a modulatory association whereby the present contextual cues exert a top-down influence over conditioned responding. Thus, when they are placed in context C they again express fear as the contextual cues in extinction are no longer present and the CS is assumed to again be predictive of shock (Bouton, 1993). Prior to the extinction test session in context C, Orsini et al. (2011) gave rats unilateral lesions (contralateral or ipsilateral) of the hippocampus and PL cortex. Rats with contralateral lesions, where the hippocampus and PL cortex are functionally disconnected, failed to exhibit the renewal of fear when the context is different from that experienced in extinction. This finding demonstrates that these animals were not capable of using the contextual cues to exhibit a renewed fear response to the extinguished conditioned stimulus. These data may suggest that the PL cortex plays a role in allowing contexts to modulate responding in an aversive setting as is the case in appetitive procedures. However, a study examining the specific role of the PL cortex in this process is lacking.

The present experiments investigated two empirical questions. Firstly, does specific inactivation of the PL cortex disrupt the ability of animals to use contextual cues to modulate responding in an aversive procedure. Secondly, does the role of the PL cortex in modulating responding on the basis of contextual cues extend to the development of an hierarchal association whereby the context comes to exert control over responding. In order to investigate these possibilities, we examined the impact of PL inactivation on the acquisition and expression of a contextual bi-conditional discrimination. This discrimination involves training rats in two different contexts with two different conditioned stimuli (CSs). In one context (context A), one CS (CS1) would be paired with shock, and the other CS (CS2) would be presented in the absence of shock. In the other context (context B), these contingencies are reversed so that CS1 would now be presented in the absence of shock, and CS2 would be paired with shock in this context. In a subsequent test session, rats will be presented with both CSs in both contexts under extinction to assess whether their behavior towards the CSs is reflective of the contexts in which they were trained. The nature of this procedure, where the CSs are predictive of shock in one context and not in another, likely necessitates that the animals use the context (for example, as a higher-order cue) to inform the animal of the status of the CS in that context. This would allow the context to exert top-down modulation over responding to the CS. Thus, investigating the role of the PL cortex in this task will bring insight into whether this region is involved in using contextual cues to modulate responding towards a CS in an aversive procedure.

We investigated the role of the PL cortex in the contextual bi-conditional discrimination at two time points. More specifically, Experiment 1 investigated the impact of PL inactivation during the extinction test session, where animals had to express the associations formed during acquisition of the contextual bi-conditional discrimination. On the basis of the research in the appetitive domain, we anticipated that animals without PL function would be unable to use the contextual cues present at test to modulate responding towards the CSs. In Experiment 2, we investigated the impact of PL inactivation during acquisition of the discrimination. If the PL cortex is involved in the development of a conditional association where the context comes to modulate the expression of a response, inactivation during acquisition will disrupt the subsequent expression of the contextually-dependent response at test.

## Materials and methods

### Subjects

Subjects were experimentally-naïve male albino Wistar rats (Laboratory Animal Services, Adelaide), weighing between 350 and 450 g at the start of the experiment. All animals were housed 4 rats per cage in a temperature- and humidity-controlled (22°C) environment in a 12 h light/dark cycle (lights on at 7:00 am). Rats were between 10–14 weeks at the commencement of the experiment. All behavioral and surgical procedures took place during the light cycle. Rats were handled 3 days prior to surgical procedures.

All animal procedures, both experimental and routine care, were carried out in accordance with the National Institute of Health Guide for the Care and Use of Laboratory Animals (NIH publications No. 80–123, revised 1996) and were approved by the University of New South Wales Animals Care and Ethics Committee (ACE: 13/102A).

### Surgical and microinfusion procedures

Prior to any training, surgery was conducted under complete anesthesia induced by inhalation of isoflurane in oxygen carrier (5% induction; 1%–2% maintenance). Following the onset of anesthesia, rats were placed in a stereotaxic frame (World Precision Instruments Inc., FL). An incision was made into the scalp, and the skin was retracted to expose the skull. For each rat, the incisor bar was adjusted such that bregma and lambda were level. Small holes above the intended lesion site were made with a high-speed dental drill, and the dura mater was severed to reveal the cortical parenchyma. Bilateral stainless steel guide cannulae (26 gauge; Plastics One, VA) were lowered 0.5 mm dorsal to the infusion site (co-ordinates relative to bregma; anteroposterior, + 3.0; mediolateral, ±0/7; dorsoventral, −3.3). Cannulae were held in place by dental cement and anchored to the skull with four fixing screws located on different bone plates. Removable dummy cannulae were inserted into the guide cannulae to prevent the cannulae from blocking.

Dummy cannulae and dust caps were removed prior to infusions. Muscimol (selective GABA_A_ agonist; 5-aminomethyl-3-hydroxyisoxazole; Sigma- Aldrich, Australia) was dissolved in nonpyrogenic saline (0.9% w/v) to obtain a final concentration of 0.5μg/μl and was infused bilaterally into the PL cortex by inserting a 33 gauge internal cannula into the guide cannula. The internal cannula was connected to a 25 μl glass syringe (Hamilton, NV) attached to an infusion pump (World Precision Instruments Inc.) and projected an additional 0.5 mm from the tip of the guide cannula. A total volume of 0.5 μl was infused bilaterally at a rate of 0.5 μl/min. This amount was in line with those used in appetitive experiments demonstrating effects selective to the PL cortex (Marquis et al., 2007). The internal cannula remained in place for an additional 60 s following infusions, allowing the bolus to be absorbed. The infusion cannulae were then removed and the dummy cannulae and dust caps replaced. The infusions occurred 10 min before the onset of the sessions where appropriate.

Rats were given 10 days to recover from surgery, after which they were placed on a food restriction schedule where they received 100 g of food pellets per cage, per day. Throughout the duration of the experiment, animals had free access to water in their home cages and were weighed three times per week to ensure they maintained at least 85% of their free-feeding weight.

### Behavioral procedures

Prior to all test sessions, rats were taken out of their home cages and transported to the testing laboratory in buckets. Rats remained in the buckets for 10 min prior to the start of the session. With the exception of the last day of training, rats received two test sessions every day, one in the morning (AM) and one in the afternoon (PM) separated by at least 3 h.

### Apparatus

Training and testing took place in 8 operant chambers (30 cm × 24 cm × 22 cm; Med Associates, VT) which were individually housed in light- and sound-attenuating compartments. Boxes were 30 cm wide × 24 cm deep × 21 cm high and consisted of two aluminium walls and an aluminium ceiling, and two Perspex side walls. The chamber floors were constructed of 19 stainless steel rods (3.8 mm in diameter, spaced 1.6 cm apart). Each chamber was equipped with a pellet dispenser that delivered one 45-mg pellet into a recessed magazine when activated. Two levers could be extended to the left and right of the recessed magazine. Two panel lights (2 cm in diameter), were located on the right hand wall of the chamber above the magazine. A 3 W house light was located on the upper left hand wall of the chambers. The chambers contained a white noise and a heavy duty relay that delivered a 5 kHz clicker stimulus. A computer equipped with MED-PC software (Med Associates) controlled the equipment and recorded the responses.

Different wallpapers, scents, and lighting were used to create two contexts that were used for the duration of the experiments. Four of the chambers were designated as contexts A (CXT A) and fitted out with laminated spotted wallpapers on three of the four walls. During sessions in these boxes, the house light and both panel lights were illuminated and diluted peppermint essence (10% solution) was dropped in four corners of the bedding below the steel rod flooring to create a strong scent within these compartments. The remaining four boxes served as context B and were fitted out with sandpaper on three of the four walls, and only the house light was illuminated during sessions in this context. Further, diluted rose essence (10% solution) was used as the defining scent for this context. Half the rats in each experiment received AM training sessions in context A and PM training sessions in context B, while the other half received AM training in context B and PM training sessions in context A.

The CSs used were 30-s presentations of a clicker stimulus and a white noise. The unconditioned stimulus (US) used for fear conditioning was a 1-s 0.8 mA shock delivered through the stainless steel rods on the floor of the operant chambers connected to a scrambled shock generator (ENV-412, Med Associates, VT). The design for Experiments 1 and 2 is represented in Table 1.

**Table 1:** **Design for Experiments 1 and 2**.

Exp	Lever training	Conditioning	Conditioning	Test
1	CXT A/CXT B	CXT A: CS1+/CS2−	CXT B: CS1−/CS2+	**CXT A/B: CS1−/CS2−**
2	CXT A/CXT B	**CXT A: CS1+/CS2−**	**CXT B: CS1−/CS2+**	CXT A/B: CS1−/CS2−

In Experiment 1 and 2, rats each received lever training in both CXT A and CXT B prior to conditioning. During conditioning sessions in CXT A, rats received pairings of CS1 with shock, while CS2 was presented without shock. In CXT B rats received pairings of CS2 with shock, while CS1 was paired without shock. Rats were then tested for levels of responding to both CSs in both CXT A and CXT B. CS1 and CS2 were either a clicker or a white noise stimulus, counterbalanced across rats. + denotes delivery of a 0.8 mA shock. Bold characters indicate infusion of either muscimol or saline.

### Instrumental training

All rats received 2 30-min sessions of magazine training, one in each context, where a 45 mg grain pellet (dustless precision grain-based pellets, Bio-serv, NJ) was delivered according to a 60-s variable time schedule. Following magazine training, rats received 6 lever-training sessions, three in each context. In context A, the right lever was made available whereas in context B the left lever was made available. In the first two lever-training sessions, each lever press was reinforced with delivery of a pellet (i.e., a CRF session) and ended once rats had received forty rewards. Rats then progressed onto a 30-s variable interval schedule where a reward became available on average once every 30 s and the next lever-press response led to the delivery of this reward. In the final two lever-training sessions, this schedule was reduced further and the reward became available on average once every 60 s. Following instrumental training, rats progressed to conditioning phase of the experiment. During all training and testing sessions, the lever was extended and reinforcement available according to a 60-s variable interval schedule as per the last sessions of training.

### Conditioning

Rats each received 7 conditioning sessions. Two CSs were used, a clicker and a white noise. If the stimulus was followed by the shock US, the shock was presented at the offset of the stimulus. For half the rats in each experiment, context A signaled that the clicker would lead to shock and the noise did not, whereas context B signaled that the noise would be followed by shock and the clicker would not. This contingency was reversed for the other half of the rats in each experiment. Each conditioning session comprised 6 CS presentations, 3 of each stimulus, with an ITI varying around a 7-min mean. This yielded sessions of approximately around 55 min long. In Experiment 1, rats received mock infusions 10 min prior to the conditioning session, where dummy cannulae were removed and the infusion needle was lowered into the PL cortex without the administration of any substance. In Experiment 2, rats received infusions of muscimol or saline into the PL cortex 10 min prior to the start of the session.

### Extinction test

Twenty four hours following the final conditioning session, rats received two extinction test sessions, one in each context. The extinction test sessions were the same as the conditioning sessions with the exception that no CS was followed by delivery of shock. In Experiment 1, rats received infusions of muscimol or saline into the PL cortex 10 min prior to the start of the extinction test session. In Experiment 2, rats received mock infusions 10 min prior to the extinction test session, where dummy cannulae were removed and the infusion needle was lowered into the PL cortex without the administration of any substance.

### Data analysis

Suppression of lever pressing was used to assess fear to the CS (i.e., according to the formula A/(A+B), where A is the period during CS presentation and B is the 30 s pre-CS period). Thus, a suppression ratio below 0.5 indicates a suppression of lever pressing during CS presentation, where lower number indicates greater suppression (i.e., greater levels of fear to the CS).

### Histology

All the end of all experiments, rats were killed with an overdose of sodium pentobarbitone (Virbac, Australia) and decapitated. Brains were removed, immediately placed on a Peltier element of a cryostat (Leica-microsystems, Australia) and frozen overnight. 40 μm coronal sections were cut through the region of the PL cortex and mounted onto glass slides. Tissue was stained using 1% cresyl violet nissl stain and subsequently assessed for the placement of cannulae microscopically by a trained observer. The PL region was defined by the boundaries specified in the atlas of Paxinos and Watson (1998). Rats with cannulae placements considered outside of the PL cortex were excluded from all analyses.

## Results

### Experiment 1: the impact of PL inactivation on the expression of the contextual bi-conditional discrimination

#### Histology

**Figure 1:**
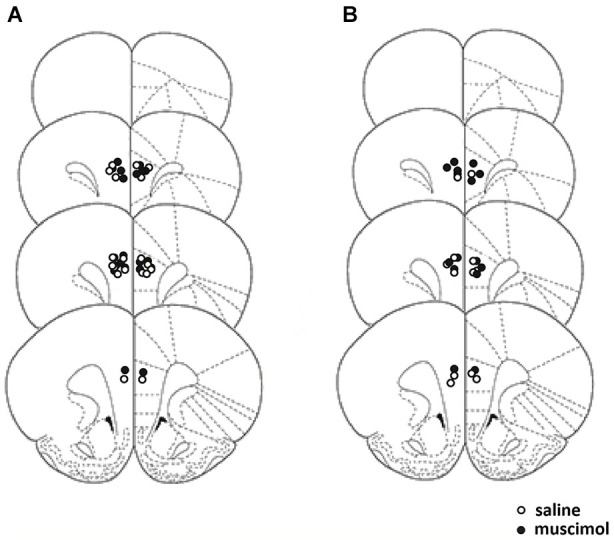
**Schematic representation of the placement of the cannula tips for Experiments 1 and 2**. Placement for cannula tips in Experiment 1 represented on the left **(A)**, and cannula placement for Experiment 2 represented on the right **(B)**. Coronal sections are taken from the following points on the antero-posterior plane beginning at top: +4.20, +3.70, +2.20, and +2.70 anterior to bregma (Paxinos and Watson, 1998).

Figure 1A illustrates the cannulae placements for rats accepted into analyses for Experiment 1. All rats recovered from surgery and no significant weight loss or behavioral problems were observed. One rat from the muscimol-infused group was found to have a cannula placement considered out of the boundaries of the PL cortex and two rats from the saline-infused group received extensive damage to the adjacent anterior cingulate cortex and so were removed from all analyses. This yielded the final group sizes, saline-infuse *n* = 9, muscimol-infused animals *n* = 12.

#### Instrumental training

All rats acquired the lever-press response within the first two CRF sessions, each receiving 40 pellets in each context. Further, all animals maintained a stable rate of responding across instrumental sessions with the leaner reinforcement schedule with a one-way ANOVA of the average number of lever presses made per minute during these sessions revealing no differences in baseline rates between the intended groups (mean (±SEM): saline 3.57 (0.49); muscimol 3.50 (0.23), *F* < 1). It is worth noting here that this is a relatively low rate of responding. It is possible that the presence of the cannulae on the heads of the animals make magazine entries slightly more arduous and therefore lever pressing is reduced as it take longer for animals to retrieve reward or check whether reward has been delivered in the magazine. This would be particularly the case early on in instrumental training when magazine entries are the dominant response. In line with this, our pilot studies have indicated that animals perform at a slightly higher rate without the presence of cannulae.

#### Conditioning

**Figure 2:**
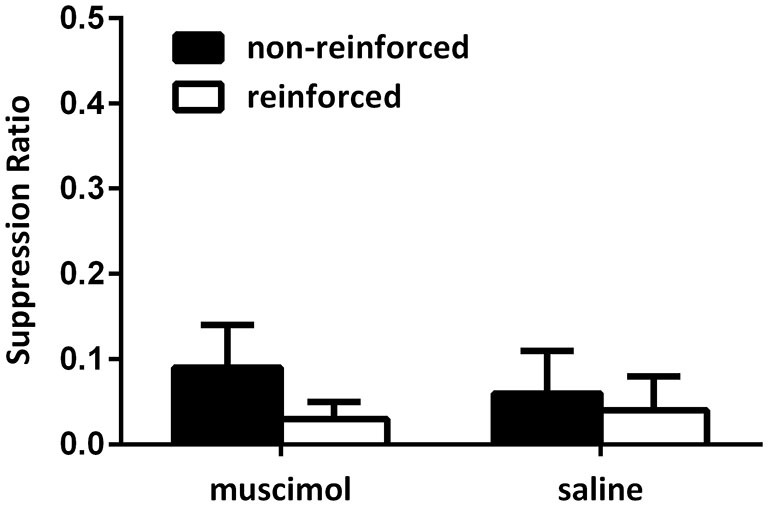
**Responding during acquisition of the contextual bi-conditional discrimination for Experiment 1**. All rats exhibited high levels of suppression towards both the reinforced and non-reinforced cues during conditioning.

Figure 2 illustrates the data from the conditioning sessions. All rats gradually suppressed their lever-pressing responses across conditioning sessions. No difference could be detected between suppression of responding toward reinforced and non-reinforced cues as animals quickly suppressed all lever-press responding during presentations of the CSs. Supporting this, a mixed-design repeated-measures ANOVA on the data from the final conditioning session revealed no main effect of CS type (mean (±SEM): to-be saline non-reinforced 0.06 (0.05) reinforced 0.04 (0.03); to-be muscimol non-reinforced 0.09 (0.05) reinforced 0.03 (0.02); *F*_(1,19)_ = 4.03, *p* > 0.05) and no interaction between the intended groups (*F* < 1). Further, this analysis also demonstrated that there was no overall differences between levels of suppression between the intended groups (*F* < 1). A one-way ANOVA also revealed that there was no difference between the intended groups in the number of lever-press responses made during the 30-s pre-CS period (mean (SEM): to-be saline 2.19 (0.37); to-be muscimol 2.72 (0.52); *F* < 1).

#### Extinction test

**Figure 3:**
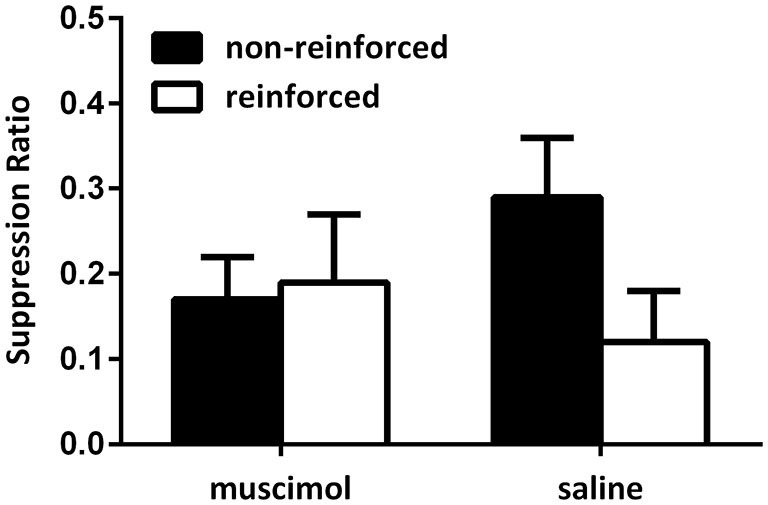
**PL inactivation during test disrupted expression of the associations acquired during the contextual bi-conditional discrimination**. Rates of responding are represented as suppression ratios for CS presentation (±SEM). Rats receiving saline infusions at test exhibited greater levels of suppression to the CS paired with shock in that context, relative to the CS presented without shock in that context. Rats receiving muscimol infusions into the PL cortex at test failed to exhibit context-specific responding towards the CSs.

Figure 3 shows the data from the critical extinction test for Experiment 1. Data were averaged across the first four trials of the first extinction test session as rats exhibited robust suppression across the first session only. These data show that animals receiving saline infusions exhibited a greater level of suppression to the CS that predicted shock in the test context, and less fear to the CS that did not predict shock in that context. Animals receiving muscimol infusions during the test session, however, failed to modulate responding towards the CS using the present contextual cues. Rather, these animals exhibited a similar level of fear to both the CS that predicted shock in the test session and the CS that did not predicted shock in that context. This was confirmed by statistical analyses. A mixed-design repeated-measures ANOVA did not reveal a significant main effect when comparing levels of responding to the CSs reinforced in the test context compared with those not reinforced in the test context (*F*_(1,19)_ = 4.01, *p* = 0.06), however, there was a significant interaction between CS and group (*F*_(1,19)_ = 7.17, *p* < 0.05). This analysis also revealed that there was no overall difference in levels of suppression between groups (*F* < 1). Follow-up analyses of simple main effects demonstrated that the source of this interaction was due to rats in the saline-infused group exhibiting a significant difference in levels of fear exhibited towards the CS trained in the test context relative to the CS that did not predict shock in that context (*F*_(1,19)_ = 9.58, *p* < 0.05). Rats infused with muscimol during the test session failed to demonstrate this difference (*F* < 1). There was no difference between groups in levels of responding to the non-reinforced cues (*F*_(1,19)_ = 1.91, *p* > 0.05), or the reinforced cues (*F* < 1). There was also no difference in average levels of pre-CS responding across these sessions (mean (±SEM): saline 3.78 (0.33); muscimol 3.63 (0.48), *F* < 1).

### Experiment 2: the impact on PL inactivation on the acquisition of the contextual bi-conditional discrimination

#### Histology

Figure 1B illustrates the cannulae placement for Experiment 2. All rats recovered from surgery and no significant weight loss or behavioral problems were observed. One rat from the muscimol-infused group had a cannula placement considered outside the PL cortex and so was excluded from all analyses. Two rats from the saline-infused group became unwell during the course of the experiment had to be removed. This yielded the final group sizes, saline-infused animals *n* = 6, muscimol-infused animals *n* = 7.

#### Instrumental training

All rats acquired both left and right lever-press responses within the first two CRF sessions, each receiving 40 pellets in each context. All animals maintained a stable rate of responding across instrumental sessions with the lean reinforcement schedule with a one-way ANOVA of the average number of lever presses made per minute during the final session demonstrating that there was no differences in lever-pressing rates between the intended groups (mean (±SEM): saline 4.52 (0.68); muscimol 4.94 (0.71), *F* < 1).

#### Conditioning

**Figure 4:**
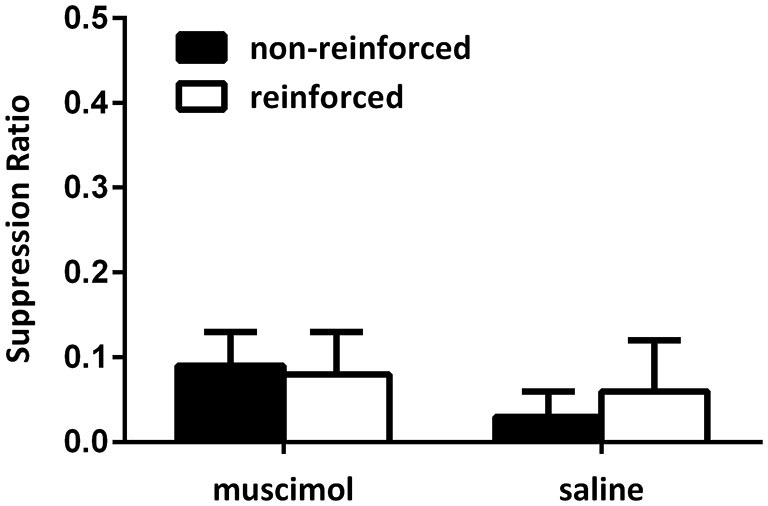
**Responding during acquisition of the contextual bi-conditional discrimination for Experiment 2**. All rats exhibited high levels of suppression towards both the reinforced and non-reinforced cues during conditioning.

Figure 4 illustrates the data from the conditioning sessions. Across conditioning sessions, all rats gradually suppressed their lever pressing responses during presentations of the CSs. Again, no difference between suppression ratios could be detected for reinforced and non-reinforced cues as animals in both groups reduced responding quickly and suppressed all responding to both stimuli during the later conditioning sessions. A mixed-design repeated-measures ANOVA of the suppression data from the final conditioning session supported this, with no main effect for CS type (i.e., non-reinforced and reinforced cues), no CS type by group interaction, and no overall between-group difference (all *F*s < 1). Further, there was no difference in average number of lever presses made during the 30-s pre-CS period across conditioning sessions (mean (±SEM): saline 2.50 (0.56); muscimol 3.07(0.29); *F*_(1,11)_ = 1.41, *p* > 0.05).

#### Extinction test

**Figure 5:**
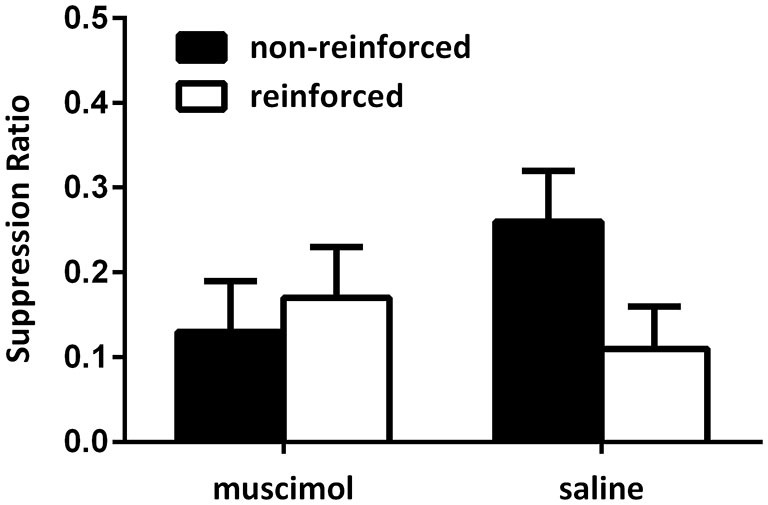
**PL inactivation during conditioning disrupted acquisition of the contextual bi-conditional discrimination**. Rates of responding are represented as mean suppression ratios (±SEM) for CS presentations. Rats receiving saline infusions during conditioning exhibited greater levels of suppression to the CS paired with shock in that context, relative to the CS presented without shock in that context. Rats receiving muscimol infusions into the PL cortex during conditioning failed to exhibit context selective responding towards the CSs.

Figure 5 shows the data from the critical extinction test session. Data were averaged across the first two trials of both extinction test sessions (i.e., the first two test trials in each context). Following the first two trials in each session, animals’ responding extinguished quickly and so the first two trials of each session provide the most informative data as to what was learnt during conditioning. These data show that saline-infused animals exhibited greater levels of fear to a CS when the CS was presented in a context where it had previously signaled shock compared to when it was presented in a context where it did not predict shock. However, infusions of muscimol into the PL cortex during conditioning disrupted the ability of animals to use contextual cues to regulate their responding to the CSs when they were tested drug free. This was confirmed with a mixed-design repeated-measures ANOVA which revealed no main effect of CS type (i.e., reinforced vs. non-reinforced cues), but a significant group by CS type interaction (*F*_(1,11)_ = 5.68, *p* < 0.05). This analysis also demonstrated that there was no significant between-group differences in overall levels of suppression (*F* < 1). Follow-up analysis of simple main effects demonstrated that the source of the interaction was due to a significant difference in responding to the non-reinforced and reinforced cues in the saline-infused animals (*F*_(1,11)_ = 7.02, *p* < 0.05), but no such difference in the muscimol-infused animals (*F* < 1). There was no significant simple main effect for responding to cues non-reinforced in the test context (*F*_(1,11)_ = 2.68, *p* > 0.05), or in responding to the cues non-reinforced in the test context (*F* < 1). Further, there were no differences in levels of lever pressing during the pre-CS period (mean (±SEM): saline 4.07 (1.52); muscimol 3.33 (0.29), *F* < 1).

## Discussion

The present experiments aimed to address two questions. Firstly, we wanted to investigate whether a role for the PL cortex in using contextual cues to modulate responding could be generalized to the aversive domain. Secondly, we wanted to examine whether the PL cortex may also be involved in the acquisition of an hierarchal association which subsequently allows an animal to use contextual cues to modulate responding. In Experiment 1 we found that animals without PL function during the extinction test session failed to use the present contextual cues to modulate responding to the CSs. Further, Experiment 2 demonstrated that the PL cortex is also involved in the acquisition of the contextual bi-conditional discrimination. That is, we found that animals without PL function during the conditioning phase failed to express context-specific responding towards the CSs when tested drug free. Taken together, these data demonstrate that the PL cortex is necessary to allow animals to modulate their response towards a stimulus. In addition, these studies show for the first time that the PL cortex also contributes to the ability of animals to form context-specific associations that allows them to use conditional cues to modulate responding towards CSs in the future.

There are many forms that the contextually-mediated associations developed in the contextual bi-conditional association may take. For example, Bouton’s (1993, 1994) influential theory of context-specific responding argues that the context comes to modulate the Pavlovian CS-US association when the meaning of the CS is rendered ambiguous. Though this theory was developed to explain the context-specificity of extinction rather than acquisition, the main principal would be that where a CS is reinforced in one context and not another, two associations are formed. One would be the excitatory CS-US association formed in sessions where the CS is reinforced, and the other the CS-no US association which comes to be regulated by present contextual cues. Thus, when the contextual cues that signal the CS will not be reinforced are present, the CS-no US association will be activated and the animal will consequently exhibit low fear towards the CS. In the case of the present study, this theory would argue that the context which signals the CS will not be reinforced will activate the CS-no US association and facilitate a decrease in fear to the cue not reinforced in that context. Thus, accordingly the PL cortex would be the site necessary to allow the context to influence activation of the Pavlovian CS-no US association.

In contrast to a role of context in exerting control over Pavlovian associations, other researchers have argued that the association that is formed when the CS is not reinforced involves a stimulus-response (S-R) association (Delamater, 1996; Rescorla, 1997). Similarly to Bouton’s (1993, 1994) theory, when the CS is reinforced this promotes the development of a Pavlovian CS-US association. However, when the CS is subsequently not reinforced, animals develop parallel inhibitory S-R associations which, when activated, reduces conditioned responding towards that CS. In line with this, the context would come to activate the inhibitory S-R association which would reduce responding towards the CS when it is presented in the context where it is not reinforced. According to this view, the PL cortex would be necessary to use the contextual cues which signal that the CS will not be reinforced to activate the inhibitory S-R which facilitates a reduction in responding when contextually appropriate. Thus, it is possible that the data garnered from the present studies is the result of contextual control over either a Pavlovian CS-US association or an inhibitory S-R association.

Given previous data have demonstrated that the PL cortex is necessary to modulate instrumental responding, it may be that the PL cortex is specifically involved in the modulation of S-R pathways as opposed to Pavlovian CS-US associations. For example, while manipulation of activity in the PL cortex disrupts the ability of animals to use the value of a goal to influence instrumental responding, this is not the case in regards to Pavlovian approach, suggesting the integrity of S-O associations remains intact in these animals (Balleine and Dickinson, 1998; Killcross and Coutureau, 2003; Haddon and Killcross, 2006). In line with this, inactivation of the adjacent infralimbic (IL) cortex reinstates goal-directed responding, presumed to be due to the dominance of PL function in the absence of the IL promotion of S-R driven habits (Tran-Tu-Yen et al., 2009). Thus, it may be that the PL cortex is specifically involved in modulating S-R associations as opposed to Pavlovian CS-US associations. In addition, there is also evidence that contextual cues can govern excitatory associations (Harris et al., 2000), thus the role for the PL cortex in allowing contextual cues to modulate responding may not be restricted to an inhibitory associations. However, further research is necessary to distinguish between these accounts.

The data from Experiment 1 are consistent with previous findings in the appetitive literature which have suggested that the PL cortex is necessary for animals to use contextual cues to modulate responding towards a CS in a rodent version of the Stroop task (Marquis et al., 2007). In this task, rats were trained on two bi-conditional discriminations, one auditory and one visual, in two distinct contexts. In one context, the two visual cues dictated pressing either the left or right lever, and in the other context the auditory cues would dictate the correct lever press (where the contextual cues are essentially incidental during this training and are not necessary to distinguish between the lever-press response that will lead to reward). The PL cortex was inactivated before a test session where animals were presented with two types of novel audio-visual compounds, congruent and incongruent, in both contexts. Congruent compounds comprised two stimuli that dictated the same lever press during training, whereas incongruent compounds comprised stimuli that dictated opposing lever presses during training. On incongruent compound trials, animals needed to use the previously irrelevant task-setting contextual cues in order to disambiguate response conflict and perform the correct lever press. Inactivation of the PL cortex before test specifically disrupted performance on the incongruent trials. The impairment is unlikely to be a consequence of a failure to process, or utilize, contextual cues to govern performance as previous research has shown that excitotoxic lesions to the prefrontal cortex (PFC) (including the PL cortex) do not impair the ability of animals to acquire context–outcome associations, or to use these associations to control responding following outcome devaluation (Haddon and Killcross, 2006). Rather, these data specifically illustrated the role of the PL cortex in using contextual cues to modulate responding towards CSs. Though some aspects of this study’s design differed from the present studies, the critical similarity is that both tasks require animals to use contextual cues to influence responding. That is, the PL cortex is only necessary when animals are required to use contextual cues to disambiguate conflict between two responses. Thus, these findings demonstrate that the PL cortex is necessary to allow animals to use contextual cues to exert control over responding towards a CS.

The rodent version of the Stroop task was designed by Haddon et al. (2008) to mimic some of the aspects of cue and response competition in the prototypical Stroop task in humans. In the prototypical Stroop task, participants are shown words that are written in different colored inks. The written words and colors can be either congruous or incongruous with one another. When subjects are shown incongruous pairs, participants show a greater number of errors when they are required to say the color of the ink as opposed to naming the word. This effect is typically attributed to the greater levels of experience in word naming over color naming, where it becomes difficult to override the tendency to name the word to accurately name the color of the word (MacLeod and Dunbar, 1988). Formally, it is argued that this occurs because the word arrives at the response stage faster than the color name. When the task requires that the subject read the word, there is no conflict as the word is processed more quickly and does not have to compete with the name of the color. However, when subjects are required to name the color, the influence of having processed the word has to be overcome before the color can be accurately named. Thus, in order to overcome the tendency to name the word, participants have to use task-setting cues (i.e., the requirement to name the color) to resolve the response conflict. This is similar to the rodent version of the Stroop task, where animals have to use the task-setting contextual cues to overcome the response conflict and perform the correct lever-press response trained in that context.

Cohen et al. (1990) developed a parallel distributed processing (PDP) model to explain the Stroop effect. Within this connectionist framework, it is proposed that populations of neurons coding for information about specific stimuli and responses become connected following repeated simultaneous activation across time. The strength of the connections between these populations determines how easily a stimulus can elicit a corresponding response and are hypothesized to occur through an associative unit, allowing these pathways to be influenced by other information external to the stimulus and response information. For example, differential task demands and contextual information can influence the level of activation of a S-R pathway. This allows relevant information to boost activation of the corresponding pathway and overcome response conflict. In the Stroop task, it is argued that participants overcome the faster processing of the word name to allow them to name the color in the incongruous case through a voluntary modulation of task demands. That is, active rehearsal of task demands (e.g., to name the ink color) is proposed to boost activation of the pathway mapping the word’s color with the response to name that color. Interestingly, later developments of this model have hypothesized that the role of the human PFC is to facilitate the increase in the appropriate S-R pathways according to current task demands (Braver and Cohen, 2000; Miller and Cohen, 2001).

In line with Cohen et al.’s (1990) model, one interpretation of the current findings is that the PL cortex may act as the site within the rodent PFC which facilitates the ability of contextual information to influence activation of the corresponding S-R pathway. In relation to the contextual bi-conditional discrimination in the present studies, the present contextual cues may influence the activation of the inhibitory S-R pathway which corresponds to the context where the CS is not reinforced. More specifically, when the CS is presented that was previously reinforced with shock in that context, the animal responds to the CS with fear as the CS-US association is activation without inhibitory influence. On the other hand, when the CS that was not reinforced with shock in the test context is presented, the present contextual cues facilitate activation of the inhibitory S-R association resulting in a reduction of the fear response in that context. This results in a comparatively greater fear response to the CS paired with shock in the test context relative to the CS presented in the absence of shock in that context. In line with a view of the PL cortex is allowing the present contextual cues to influence activation of an inhibitory S-R pathway, the data from Experiment 1 demonstrates that rats without PL function are not capable of using the present contextual cues to influence fear responding. Rather, we found that PL inactivation during the test session resulted in animals exhibiting equivalent levels of fear to both CSs in either context. This view involves assuming that this particular task involved the formation of inhibitory S-R associations (Delamater, 1996; Rescorla, 1997) and allows us to reconcile the current data with the wider literature investigating PL function.

Significantly, the data from Experiment 2 demonstrated that the integrity of function in the PL cortex is also necessary during acquisition of the discrimination. That is, in Experiment 2 we found that animals without PL function during the conditioning session were not capable of using the contextual cues present during the test session to influence fear responding. For the first time, these data show that the PL cortex is not only involved in the ability of animals to modulate performance on the basis of present contextual cues but also to facilitate development of the context-specific associations. In line with the framework being developed, we would interpret these data as reflecting a role for the PL cortex in the development of a hierarchal network that allows the inhibitory S-R pathway to become connected to the context in which it was trained, subsequently enabling context-specific responding. This is the first demonstration of a role for the PL cortex in the development of context-specific associations, allowing the present experiments to expand the theories of the function of the PL cortex to a role for this region in the learning process.

The PL cortex is a part of the larger medial prefrontal region in rodents. Adjacent to the PL cortex is the IL cortex which has been implicated in a different aspect of the rodent Stroop task developed by Haddon et al. (2008). Interestingly, the IL cortex appears to function in a manner that opposes the influence of the PL cortex. For example, Haddon and Killcross (2011) examined the impact of IL inactivation on the rodent version of the Stroop task found to require the PL cortex (Marquis et al., 2007). In this study, the amount of training on each of the conditional discriminations (i.e., two distinct visual stimuli predict which lever-press will be reinforced in one context, with two auditory stimuli dictating the contingencies in another) was manipulated such that one discrimination received three times the training of the other. As a consequence of this differential training, and in line with the prototypical Stroop task in humans, rats show greater interference from the overtrained response when given an incongruent compound in the undertrained context. That is, animals are unable to use the undertrained task-setting contextual stimuli to resolve the conflict when these contextual stimuli have to dominate the overtrained response. However, when the IL cortex was inactivated prior to test, animals were rendered more able to use the contextual stimuli relevant to the undertrained response to overcome the overtrained response. Thus, in contrast to the role of the PL cortex in facilitating the use of contextual cues during response conflict, the IL cortex seems to be involved in attenuating the influence of these cues in favor of allowing a well-trained context-independent response to dominate behavior, independently of contextual cues.

There is also data in the aversive domain that suggests the IL cortex plays a role in generalizing responding across contexts. For example, Quirk et al. (2000) found that IL lesions produce a greater level of spontaneous recovery following fear extinction. Animals were first trained to fear a CS presented with shock. Following conditioning, the CS was presented in the absence of shock and level of fear towards the CS were extinguished. On the following day animals were tested for levels of fear recovery when the CS was presented. Animals with IL lesions exhibited a significantly greater level of fear towards the extinguished CS relative to sham-lesioned animals. Given the recovery of fear has also been interpreted as the result of animals detecting a change in temporal context (Bouton, 1993, 1994), these data are consistent with a role for IL in generalizing responding across contexts. More recently, this view has been supported with an ABA renewal paradigm where IL lesions produce a greater level of renewal of fear following a physical change in context (Zelikowsky et al., 2013), consistent with findings in the appetitive literature which have also demonstrated higher levels of renewal in an ABA renewal paradigm in an appetitive procedure (Rhodes and Killcross, 2007). These data support a role for the IL cortex in opposing a process where the PL cortex promotes context-specific responding, and suggest that the role of the IL cortex in this process generalizes across the appetitive and aversive domains.

In line with the framework discussed above, these data can be interpreted as a role for the IL cortex in opposing the process whereby the PL cortex exert top-down control to bias activation of the S-R pathway appropriate to current circumstance. Rather, the IL cortex appears to promote the performance of the response at the terminal end of the stronger S-R pathway. This results in behavior becoming impervious to the influence of contextual cues following extended training, where the strong S-R pathway becomes capable of dominating behavior. It is unclear whether the IL cortex directly opposes the influence of the PL locally, or whether the IL cortex promotes the activation of the stronger S-R pathway at a broader, circuit level. Given the lack of projections of the IL cortex to other regions involved in promoting habitual performance (i.e., the dorsolateral striatum, DLS; but see a role for the central nucleus of the amygdala, Lingawi and Balleine, 2012), it may be that the role of the IL cortex is to locally inhibit activation of the PL cortex, indirectly modulating the influence of task-setting contextual information on performance. This account is supported by evidence demonstrating the reciprocal nature of the interactions between the PL and IL cortices (Killcross and Coutureau, 2003; Vidal-Gonzalez et al., 2006). These data suggest that controlled processing may be achieved not only by the boosting the ability of these task-setting cues to modulate responding, but also reducing the effectiveness of those cues to influence behavior following extensive experience through an active promotion of the stronger S-R pathway. As a final point, it is worth noting here that we do not argue against a role for inhibitory processes *per se* (as is the dominant view in the fear conditioning literature), rather, the IL cortex is argued to be involved in the promotion of S-R pathways independently of contextual cues, whether the associations between the stimulus and response is inhibitory or excitatory. For example, in the case of extinction these S-R pathways may involve inhibitory associations (Delamater, 1996; Rescorla, 1997), however, in the case of appetitive habits this is unlikely to be the case. Either way, the critical notion is that the IL cortex is important for promoting the dominance of these associations as reflected in behavior.

The present experiments have demonstrated that the PL cortex is involved in facilitating the use of contextual cues to modulate responding towards a CS in the aversive domain. Further, we have found for the first time that this region is involved in the formation of the context-specific associations. In doing so, we have found evidence to support for a framework whereby the PL cortex exerts top-down control over S-R pathways according to present contextual cues, to influence both the acquisition and expression of conditioned fear. This is consistent with the functional role of the PL cortex in appetitive procedures. In contrast, previous research has suggested that the IL cortex opposes the role of the PL cortex in facilitating context-specific responding to promote execution of the well-trained, context-independent response across both aversive and appetitive procedure. Taken together, these data suggest that the PL and IL cortices act in a manner to establish a trade-off between using contextual cues to change responding when appropriate and performing a well-trained response independently of contextual cues.

## Conflict of interest statement

The authors declare that the research was conducted in the absence of any commercial or financial relationships that could be construed as a potential conflict of interest.
